# Association between Cognitive Reserve Indicator and Chronic Disease-Free Survival: A Large Community-Based Longitudinal Study

**DOI:** 10.14283/jpad.2024.160

**Published:** 2024-09-18

**Authors:** Pengcheng Li, Wenzhe Yang, J. Wang, Hong Zhu, Abigail Dove, Weili Xu

**Affiliations:** 1 Department of Epidemiology and Biostatistics, School of Public Health, Tianjin Medical University, Qixiangtai Road 22, Heping District, 300070 Tianjin, P.R. China; 2 Tianjin Key Laboratory of Environment, Nutrition, and Public Health, Tianjin Medical University, Tianjin, China; 3 Center for International Collaborative Research on Environment, Nutrition and Public Health, Tianjin Medical University, Tianjin, China; 4 Department of Epidemiology, College of Preventive Medicine, Army Medical University (Third Military Medical University), Chongqing, China; 5 Aging Research Center, Department of Neurobiology, Care Sciences and Society, Karolinska Institutet, Tomtebodavägen 18A Floor 10, Solna, SE-171 65 Stockholm, Sweden

**Keywords:** Cognitive reserve, chronic disease, longevity, UK biobank

## Abstract

**Background:**

Cognitive reserve (CR) has been linked to dementia and might be a predictor of aged-related outcomes. However, the association between CR and risk of other chronic diseases and mortality remains unclear.

**Objectives:**

We aimed to investigate the association of CR with survival free from major chronic diseases.

**Design, Setting and Participants:**

This community-based longitudinal study used data from the UK Biobank. A total of 412,509 participants (mean age 55.71±8.10) free of major chronic disease (including dementia, diabetes, cardiovascular diseases, chronic obstructive pulmonary disease, and cancer) completed the baseline examination between 2006 to 2010 and were followed for changes in health status.

**Measurements:**

Latent class analysis was used to generate an indicator of CR (categorized as low, moderate, or high) based on education, occupation, television viewing time, confiding, social connection, and leisure activities. Major chronic diseases and survival status were ascertained through self-reported history and/or linkages to medical and death records. Chronic disease-free survival was defined as survival without any of the aforementioned chronic diseases. Effect modifications and interactions between the CR indicator and sex, age, and lifestyle factors (including smoking status, alcohol consumption, physical activity, and body mass index) were explored.

**Results:**

Over a median follow-up of 12.49 (interquartile range 11.42–13.41, range 0.01–15.87) years, 112,190 (27.2%) participants died or developed at least one chronic disease. High CR indicator was associated with lower risk of chronic disease/death (hazard ratio 0.82, 95% confidence interval: 0.80–0.83) compared to low CR indicator. Chronic disease-free survival was prolonged by 1.33 (1.21–1.44) years among participants with high CR compared to low CR indicator. Furthermore, the association between the CR indicator and chronic disease-free survival was strengthened among individuals aged <60 years and current smokers.

**Conclusion:**

High CR indicator is associated with a lower risk of chronic disease/death and may prolong chronic disease-free survival. Our findings underscore the importance of CR-enhancing lifestyle and experiences in health longevity, especially for younger individuals and current smokers.

**Electronic Supplementary Material:**

Supplementary material is available in the online version of this article at 10.14283/jpad.2024.160.

## Introduction

The average human life expectancy has increased by about 23 years over the past few decades (1). However, not all of these additional years of life are spent in optimal health (2, 3). Recent Global Burden of Disease studies have highlighted that with age, middle-aged and older adults live more years with various chronic diseases, especially dementia, diabetes, cardiovascular diseases (CVDs), chronic obstructive pulmonary disease (COPD), and cancer, all of which are among the top ten contributors to reduced quality of life and premature death (3, 4). These diseases impose a considerable burden on patients, families, and society as a whole (5). Therefore, a relevant issue is how to prevent these major chronic diseases and maximize lifespan lived in good health (6).

Cognitive reserve (CR) is a construct in dementia research that refers to the hypothesis that lifelong engagement in stimulating activities can enhance the brain’s capacity to withstand age- and pathology-related damage (7). CR is commonly measured using proxy indicators like education level, occupational attainment, and engagement in social, cognitive, or leisure activities (8, 9). Combining multiple proxies could be create a more robust indicator of CR (10). High CR has been linked to reduced risks of dementia and cerebral small vessel disease (11, 12) possibly through mechanisms including neurogenesis, oxidative stress, inflammation, and energy metabolism which are also involved in the development of cardiometabolic disease, lung disease, and cancer (13–16). Thus, it is plausible that CR might play a role in these other chronic diseases.

Beyond morbidity and mortality, it is crucial to consider measures that reflect healthy life span and quality of life. Disease-free survival is one such measure (17–19) that has been increasingly used in public health (17, 20, 21). To our knowledge, only one study up to now has investigated an association between CR and death, suggesting that CR level (measured by cognitive tests) is negatively related to total mortality (22). Furthermore, several studies have shown that individual CR-related factors (including education (23), occupation (24), social/leisure activity engagement (25, 26), and television (TV) viewing (25)) are associated with major chronic disease-free survival. However, the above studies have only focused on single proxies for CR and specific diseases, and the relationship between a composite measure of CR comprised of multiple related factors and chronic disease-free survival has not been investigated yet. In addition, given that age, sex, and lifestyle behaviors can modify the risk of chronic disease and mortality (27, 28), exploring potential effect modifiers may help identify populations that stand to benefit most from targeted prevention strategies.

Based on the current evidence, we hypothesized that a high level of CR is associated with a reduced risk of major chronic diseases/death and prolonged chronic disease-free survival, and that sociodemographic and lifestyle factors may modify these associations. In this study, we aimed to verify the hypotheses using longitudinal data from the UK Biobank.

## Methods

### Study population

Data were derived from the UK Biobank (Application Number 67048), a community-based prospective study consisting of >500,000 participants aged 37 to 73 years recruited from 22 sites across the UK. Between 2006 and 2010, 502,412 individuals participated in the baseline survey and completed a series of sociodemographic, physical, peripheral blood, and medical assessments. Of these participants, we excluded 88,974 with prevalent chronic diseases (i.e., dementia, diabetes, CVDs, COPD, and cancer) at baseline and 929 with missing information on the timing of chronic disease diagnosis, leaving a study sample of 412,509 participants (eFigure 1).

All participants provided informed and written consent. The UK Biobank study received ethical approval from the North West Multi-Centre Research Ethics Committee (21/NW/0157).

### Data collection

Information on sociodemographic and lifestyle factors were collected through computerized touch-screen questionnaires at baseline. Race was dichotomized as white vs. non-white. Smoking and alcohol status were categorized as never, previous, or current smoking/drinking. Physical activity was measured in terms of metabolic equivalents (MET) per week using the International Physical Activity Questionnaire short form and classified as inactive (<600 MET-min/week), moderate (600 to <3000 MET-min/week), or active (≥3000 MET-min/week) (29). Body mass index (BMI) (kg/m^2^) was calculated as weight (kg) divided by the square of height (m). Hypertension was ascertained based on systolic blood pressure ≥140 mm Hg, diastolic blood pressure ≥90 mm Hg, self-reported history of hypertension, use of antihypertensive drugs, or medical records.

### Assessment of CR indicator

Education, occupational attainment, TV viewing time (regarded as cognitively passive sedentary behavior (30)), frequency of confiding, frequency of social connection, and richness of leisure activity engagement reflect different aspects of CR, as defined in previous studies (12, 31, 32), and thus we used these six variables assessed based on participants’ self-reports at baseline to generate an overall CR indicator.

Education was classified as five levels based on the number of years corresponding to qualifications (33), including 1) no educational qualifications, 2) Certificate of Secondary Education, O levels/General Certificate of Secondary Education or equivalent, 3) A levels/AS levels, other professional qualifications or equivalent, 4) National Vocational Qualification, Higher National Diploma, Higher National Certificate or equivalent, and 5) college/university degree.

Occupational attainment was assessed based on employment status and job titles. Each self-reported job title was matched to a 4-digit job code derived from the Standard Occupational Classification 2000 system, which was developed by the UK Office of National Statistics (34). A socio-economic classification (SEC) was established based on the job codes, and was scored as 1 to 7, with lower values reflecting higher socio-economic position. Therefore, the SEC, a clear ordinal variable, was used to represent the occupational attainment (35, 36). These categories of occupational attainment included 1) unemployed or SEC 7 (routine occupations), 2) SEC 4–6 (small employers and own account workers, lower supervisory and technical occupations or semi-routine occupations), 3) SEC 3 (intermediate occupations), 4) SEC 2 (lower managerial and professional occupations), and 5) SEC 1.1 (large employers and higher managerial occupations) or 1.2 (higher professional occupations).

TV viewing time was classified as four levels based on the total hours of daily TV watching, including 1) ≥4, 2) 3–3.9, 3) 2–2.9, and 4) <2.

Frequency of confiding was categorized into four levels based on the frequency of participants confiding in someone close to them, including 1) never or almost never, 2) about once a month or less, 3) 1–4 times a week, and 4) almost daily.

Frequency of social connection was classified into three levels based on the frequency of participants making or receiving friend/family visits, including 1) about once a month or less, 2) about once a week, and 3) 2–4 times a week or more.

Richness of leisure activity engagement was divided into three levels based on the total number of types of activities in which participants attended once a week or more often, including 1) 0, 2) 1, and 3) 2–5. These activities included sports club or gym, pub or social club, religious group, adult education classes, or other group activity.

The CR indicator was created using latent class analysis (LCA), which can identify hidden clusters by grouping multiple observed variables into a latent variable with mutually exclusive latent classes (37). We identified a three-latent-class model as having the best fit based on model selection (relatively low Bayesian information criterion) and the uncertainty of posterior classification (all mean posterior probabilities in three latent classes ≥0.70). Latent class 1 (high CR indicator; n=168,211 [40.8%]) was characterized by a high level of CR-related factors, specifically, with higher levels of education, occupational attainment, confiding, and leisure activities as well as less time spent watching TV. Latent class 2 (moderate CR indicator; n=167,766 [40.7%]) and latent class 3 (low CR indicator; n=76,532 [18.5%]) were characterized by moderate and low levels of CR-related factors in general, respectively. More detailed descriptions of the LCA are available in eMethod 1.

### Assessment of chronic diseases and death

We focused on five major chronic diseases, including dementia, diabetes, CVDs, COPD, and cancer. Dementia, COPD, and CVDs (including heart disease [ischemic heart disease, atrial fibrillation, and heart failure] and stroke) were ascertained based on information from self-reported medical history, medical and death records. Diabetes was considered if the presence of hemoglobin A1c ≥6.5%, fasting plasma glucose ≥126 mg/dl, self-reported history of diabetes, use of glucose-lowering medications, or medical records. Cancer was assessed through self-reports, medical and death records, and the cancer register. Codes used to identify chronic diseases are listed in eTable 1. Deaths from all causes were identified from death records. Chronic disease-free survival was defined as the years lived until the occurrence of any chronic disease or death.

### Statistical analysis

Baseline characteristics of the study population by CR level were tested using *χ*^2^ test for categorical variables and one-way analysis of variance followed by Bonferroni correction for continuous variables. Missing values for the following variables were imputed using multiple imputation by chained equations: education (n=7903 [1.9%]), occupation (n=74,851 [18.2%]), TV viewing time (n=4011 [1.0%]), confiding (n=14,894 [3.61%]), social connection (n=6330 [1.5%]), leisure activities (n=2177 [0.5%]), sex (n=1 [<0.01%]), race (n=2199 [0.5%]), smoking status (n=2200 [0.5%]), alcohol consumption (n=1271 [0.3%]), physical activity (n=80,134 [19.4%]), and BMI (n=2276 [0.6%]).

Cox proportional hazard regression models were applied to examine the associations of different levels of CR with the outcomes of interest. Follow-up time was calculated as the time from baseline until death or the end of follow-up (January 20, 2022). A combined outcome was defined as either incident chronic disease or death. Follow-up time was calculated as the time from baseline until the occurrence of any chronic disease, death, or the end of follow-up, whichever occurred first. The proportional hazard assumption was assessed using Schoenfeld residuals, and no violations were observed. Multi-state Markov model was used to further assess the effect of the CR indicator on death among participants with incident chronic diseases. The model included three states (chronic disease-free, chronic disease, and death) and three transitions: 1) from chronic disease-free to incident chronic disease, 2) from chronic disease-free to death, and 3) from incident chronic disease to death (eFigure 2).

We then used Laplace regression to estimate the absolute difference in the median time until chronic disease onset or death by CR level (i.e. years of chronic disease-free survival) (38). Considering that 4.7% and 27.2% of participants died and experienced chronic disease/death, respectively, we assessed the percentile differences (PDs) in time (years) to death and chronic disease/death for the first 10% and first 33% of participants, respectively. We also assessed the association between the CR indicator and chronic disease-free survival separately for each included chronic disease.

Finally, to further assess effect modification and interaction, we explored multiplicative interactions between the CR indicator and sex, age (<60 vs. 60+), and lifestyle factors (including smoking status [never/previous vs. current], alcohol consumption [never/previous vs. current], physical exercise [inactive vs. moderate/active], and BMI [<25 vs. ≥25 kg/m2]) on chronic disease-free survival, followed by stratified analyses by those factors. In addition, we examined the joint effect of the CR indicator and above potential modifiers on the outcomes, where additive interaction was evaluated by computing the relative excess risk due to interaction (RERI), the attributable proportion (AP), and the synergy index (SI).

In supplementary analyses, we repeated analyses after 1) excluding participants with missing information (n=147,920), 2) excluding participants who developed incident chronic disease or died in the first two years from baseline (n=13,985) to reduce the possibility of reverse causality, and 3) removing dementia from the chronic disease outcome because of the established CR-dementia association.

All analyses were first adjusted for age, sex, and race (basic-adjusted models) and then further adjusted for smoking status, alcohol consumption, physical activity, BMI, and hypertension (multi-adjusted models). All P-values were two-tailed, and statistical significance was defined as P <0.05. Analyses were performed using R, version 4.3.0 and STATA SE 15.0 (StataCorp, College Station, TX, USA).

## Results

### Characteristics of the study population

Of the 412,509 chronic disease-free participants (mean age 55.71±8.10 years), 233,184 (56.5%) were female. Compared to participants with a low level of CR, those with moderate or high level of CR were more likely to be younger, never smokers, drinkers, physically active and to have lower BMI and a history of hypertension (Table 1).

**Table 1:** Baseline characteristics of the participants by different levels of cognitive reserve

**Characteristics**	**Cognitive reserve**			**P-value**
	**Low (n=76532)**	**Moderate (n=167766)**	**High (n=168211)**	
Age (year)	59.19±7.38	55.34±8.10	54.49±7.98	<0.001
Female	43370 (56.7)	100107 (59.7)	89707 (53.3)	<0.001
Race-white	71031 (92.8)	156044 (93.0)	147648 (87.8)	<0.001
Smoking status				<0.001
Never	36860 (48.2)	94407 (56.3)	103309 (61.4)	
Previous	27736 (36.2)	55433 (33.0)	52219 (31.0)	
Current	11936 (15.6)	17926 (10.7)	12683 (7.5)	
Alcohol consumption				<0.001
Never	4936 (6.4)	6440 (3.8)	5673 (3.4)	
Previous	3792 (5.0)	4815 (2.9)	4221 (2.5)	
Current	67804 (88.6)	156511 (93.3)	158317 (94.1)	
Physical activity				<0.001
Inactive	13763 (18.0)	29760 (17.7)	30424 (18.1)	
Moderate	33463 (43.7)	80884 (48.2)	94122 (55.9)	
Active	29306 (38.3)	57122 (34.1)	43665 (26.0)	
Body mass index (kg/m^2^)	28.07±4.82	27.38±4.65	26.39±4.31	<0.001
Hypertension	24736 (32.3)	40830 (24.3)	33720 (20.1)	<0.001

Note. Data are presented as mean ± standard deviation or number (%).

### Association of CR with incident chronic diseases and death

During the follow-up (median 12.49 years, interquartile range 11.42–13.41 years, range 0.01–15.87 years), 112,190 (27.2%) participants died or developed incident chronic disease. Of the 109,802 participants with any incident chronic disease, 16,807 (15.3%) died before the end of follow-up. In the multi-adjusted Cox models, both moderate and high CR indicator were associated with lower risk of all-cause mortality (HR 0.76, 95% CI: 0.74, 0.79; HR 0.71, 95% CI: 0.69, 0.74) and chronic disease/death (HR 0.87, 95% CI: 0.85, 0.88; HR 0.82, 95% CI: 0.80, 0.83) compared to low CR indicator. In the multistate model, among participants with incident chronic diseases, moderate/high CR indicator was associated with decreased mortality (HR 0.91, 95% CI: 0.87, 0.94; HR 0.90, 95% CI: 0.87, 0.94) compared to low CR indicator (Table 2).

**Table 2:** Hazard ratios (HRs) and 95% confidence intervals (CIs) of incident outcomes (death and/or chronic disease) and death among participants with incident chronic diseases in relation to the cognitive reserve indicator

**Cognitive reserve**	**No. of subjects**	**No. of cases**	**Basic-adjusted HR (95% CI)**^**a**^	**Multi-adjusted HR (95% CI)**^**b**^
Death				
Low	76532	6092	1.00 (Reference)	1.00 (Reference)
Moderate	167766	7092	0.70 (0.68, 0.73)	0.76 (0.74, 0.79)
High	168211	6011	0.61 (0.59, 0.63)	0.71 (0.69, 0.74)
Chronic disease/Death				
Low	76532	29109	1.00 (Reference)	1.00 (Reference)
Moderate	167766	44305	0.82 (0.81, 0.83)	0.87 (0.85, 0.88)
High	168211	38776	0.72 (0.70, 0.73)	0.82 (0.80, 0.83)
Death among participants with incident chronic disease				
Low	28400	5383	1.00 (Reference)	1.00 (Reference)
Moderate	43421	6208	0.86 (0.84, 0.90)	0.91 (0.87, 0.94)
High	37981	5216	0.84 (0.81, 0.87)	0.90 (0.87, 0.94)

a. Models were adjusted for age, sex, and race; b. Models were further adjusted for smoking status, alcohol consumption, physical activity, body mass index, and hypertension.

### Association of CR with chronic disease-free survival

In multi-adjusted Laplace regression, moderate and high CR indicator prolonged survival by 1.47 (10th PD, 95% CI: 1.25, 1.68) years and 1.80 (10th PD, 95% CI: 1.57, 2.03) years, respectively (eTable 2). Furthermore, when incident chronic disease and death were considered as a combined outcome, participants with a moderate (33th PD 0.92, 95% CI: 0.81, 1.03) or a high (33th PD 1.33, 95% CI: 1.21, 1.44) level of CR had longer chronic disease-free survival than those with a low level of CR (Table 3).

**Table 3:** The 33th percentile differences (PDs) and 95% confidence intervals in time (years) to chronic disease/death in relation to the cognitive reserve indicator

**Cognitive reserve**	**No. of subjects**	**Chronic disease/Death**		
		**No. of cases**	**Basic-adjusted 33th PD (95% CI)**^**a**^	**Multi-adjusted 33 th PD (95% CI)**^**b**^
Low	76532	29109	0.00 (Reference)	0.00 (Reference)
Moderate	167766	44305	1.45 (1.34, 1.57)	0.92 (0.81, 1.03)
High	168211	38776	2.35 (2.23, 2.46)	1.33 (1.21, 1.44)

a. Models were adjusted for age, sex, and race; b. Models were further adjusted for smoking status, alcohol consumption, physical activity, body mass index, and hypertension.

Regarding individual chronic disease, moderate and high CR indicator were also associated with 0.46 to 2.84 years of longer survival free of dementia, diabetes, CVDs, COPD, or cancer (eTable 3).

### Effect modification and interaction

**Figure 1:**
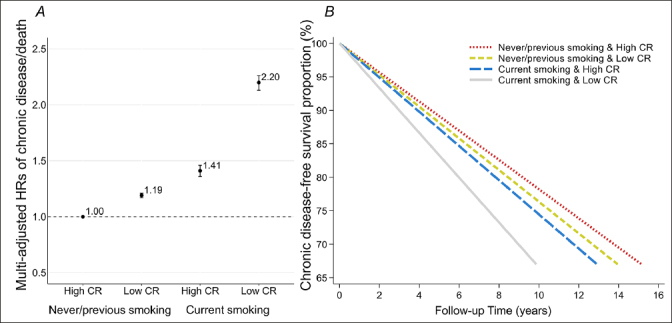
Hazard ratios (HRs) from Cox models (A) and 33th percentile differences (PDs) in time (years) to incident outcomes (chronic disease/death) from Laplace regression (B), and 95% confidence intervals (CIs) in relation to joint exposure of smoking status and the cognitive reserve indicator Note. Models were adjusted for age, sex, race, alcohol consumption, physical activity, body mass index, and hypertension. Measures of additive interaction between smoking status and the cognitive reserve indicator on chronic disease/death: RERI: 0.60, 95%CI: 0.53–0.68, P<0.001; AP: 0.27, 95%CI: 0.25–0.30, P<0.001; SI: 2.02, 95%CI: 1.83–2.23, P<0.001.

We observed significant effect modification of the association between the CR indicator and chronic disease-free survival according to age group and smoking status (both P<0.001). Specifically, higher CR indicator was associated with lower risk of chronic disease/death and longer chronic disease-free survival to a larger extent in participants aged <60 years than in those aged 60+ years (eTable 4). Moreover, the effect of CR on chronic disease-free survival was strengthened among current smokers relative to never/previous smokers (eTable 5). In analysis of joint effect of CR and smoking, compared to never/previous smokers with high CR indicator, current smokers with low CR indicator had excess higher risk of chronic disease/death (HR 2.20, 95% CI: 2.13, 2.26) and much shorter chronic disease-free survival (33th PD −5.13, 95% CI: −5.32, −4.93). There was a significant additive interaction between smoking status and the CR indicator on chronic disease/death: RERI of 0.60 (95% CI: 0.53, 0.68) indicating that the combined effect of smoking and low CR on the outcome was greater than the sum of the individual effect of the two factors, AP of 0.27 (95% CI: 0.25, 0.30) indicating that 27% of chronic disease/death was due to an additive interaction, and SI of 2.02 (95% CI: 1.83, 2.23) indicating a synergistic effect of the two exposures on the outcome (Figure 1 and eTable 6). The difference in HRs between high CR indicator combined with smoking and low CR indicator combined with smoking was significant (HR 0.63, 95% CI: 0.61, 0.66), indicating that the risk of chronic disease/death related to current smoking was diminished by 37% by high CR indicator.

However, no significant multiplicative or additive interaction was observed between the CR indicator and sex, alcohol consumption, physical activity, or BMI on chronic disease-free survival (all P >0.05) (eTables 7–10).

### Supplementary analysis

Similar results were obtained when we 1) excluded individuals with missing data (eTable 11), 2) excluded those with incident chronic disease/death within the first two years of follow-up (eTable 12), 3) removed dementia from the chronic disease outcome (eTable 13).

## Discussion

In this longitudinal study of >400,000 participants, we found that high CR indicator might reduce the risk of chronic disease/death by about 20% and prolong chronic disease-free survival by 1.3 years. These associations appeared to be strengthened among participants aged <60 years and current smokers, and the risk effect of smoking on chronic disease-free survival was reduced among those with high CR indicator.

Evidence suggests that some single CR-related factors including higher education level (39), higher occupational attainment (40), minimal TV watching (41), actively engaging in leisure activities (42), and more frequent social contact (42), might be protective against chronic diseases. Although these studies focused on different chronic diseases, they all included CVDs, diabetes (39), and cancer (41), with others additionally focusing on dementia (42) and COPD (40). However, no study to date has comprehensively examined the association between CR and major chronic diseases. Furthermore, only one cohort study related higher CR (measured by cognitive tests) to lower mortality risk (22). In the present study, we used LCA to create a comprehensive CR indicator reflecting a range of mentally- and socially- stimulating experiences across the life course, finding that a higher level of CR was associated with reduced risk of both major chronic diseases and death, even among people with chronic diseases.

Compared to mortality, disease-free survival is a better indicator of healthy aging that reflects healthy lifespan. A previous cohort study showed that more highly educated individuals lived longer without longstanding illness (43). In a multi-cohort study, participants with higher occupational attainment were expected to survival longer free of heart disease, stroke, chronic lung disease, and cancer (24). A sub-study of Cardiovascular Health Study also suggested that more frequent social connection (such as social network and social support) was related to longer life expectancy and disability-free life expectancy (26). Additionally, less TV viewing has been linked to more years lived free of colorectal, lung, and postmenopausal breast cancer (25). Notably, previous studies used a variety of definitions of CR based on data availability and mainly focused on survival/life expectancy free of specific chronic diseases, making it challenging to directly compare the findings across studies. The present study takes the further step of examining the relationship between a composite indicator of CR and survival free from major chronic diseases. Specifically, we observed that high CR indicator might prolong survival free from dementia, diabetes, CVDs, COPD, and cancer by almost 1.4 years. Together, our findings emphasize the potential of CR to not only the prevention of chronic disease but also promotion of healthy longevity.

We further explored age, sex, and smoking as potential effect modifiers. We identified an apparently stronger association between the CR indicator and chronic disease-free survival among younger (<60 years) compared to older (60+ years) participants. This may be because younger adults already have a longer life expectancy than older adults (1), and therefore may have more potential to benefit from enhanced CR to minimize the risk of chronic disease and premature death. We also observed an additive interaction between smoking and the CR indicator on outcomes: current smokers with low CR indicator had an excess higher risk of chronic disease/death and much shorter chronic disease-free survival compared to never/previous smokers with high CR indicator. Notably, nearly 40% of the risk of chronic disease/death related to smoking was diminished and 3 years of the duration of chronic disease-free survival related to smoking was prolonged by high CR indicator. That is to say, enhancing the accumulation of CR might mitigate the detrimental effect of smoking on chronic disease-free survival. However, we did not find any difference according to sex or other lifestyle factors, and further studies are warranted to investigate potential modification effects. Our findings have important public health implications, as CR-promoting experiences and behaviors may not only benefit healthy aging for the general population but also be more effective when targeted at younger individuals and current smokers.

Several mechanisms could explain the association between the CR indicator and chronic disease-free survival. Biologically, CR is hypothesized to work through neural reserve (i.e. higher intrinsic brain network connectivity that is better able to tolerate brain pathology) and/or neural compensation (i.e. fostering the development of alternative neural pathways that take over the tasks performed by damaged ones) to protect against neurodegenerative disorders (7, 44). Enhanced CR can help maintain or improve neuronal density and connectivity which are also important for the tumor microenvironment related to the development of many cancers, such as breast, prostatic, pancreatic, gastric and cerebral cancers (45, 46). Furthermore, in animal studies, enriched environments (characterized by novel stimuli; analogous to the accumulation of CR in humans) (47), contribute to improving neural plasticity and neuroendocrine function (48), increasing levels of brain-derived neurotrophic factor, counterbalancing immune response, and reducing inflammation (49) and oxidative stress (50), which all play a protective role against the development of cardio-cerebral vascular disease, endocrine metabolic disease, cognitive impairment, chronic lung disease, and cancer (13–16). In addition, we observed a higher prevalence of hypertension among participants with low CR indicator, suggesting that vascular pathologies might explain the CR-CVDs association. Behaviorally, individuals with a higher level of CR (i.e. higher educational and occupational attainment) are more likely to have higher socioeconomic status, greater health literacy, better access to medical services, and healthier lifestyle (such as avoiding smoking) (23, 51), which could contribute to lower chronic disease and mortality risk. Sedentary behavior (e.g. TV viewing) is associated with incident diabetes, CVDs, COPD, and total mortality partly through decreasing insulin sensitivity and blood circulation, promoting oxidative stress, and impairing vascular endothelial dilation (52, 53). Social engagement may promote healthy longevity by increasing an individual’s access to psychological support and promoting health-related behavior (54, 55).

A notable strength of this study is the use of LCA to construct a composite CR indicator that captures the accumulation and interaction of multiple CR-related factors. Additionally, our outcome, chronic disease-free survival outcome, is especially relevant for public health as it reflects not only lifespan but also healthspan. Nevertheless, several limitations should be acknowledged. First, although diseases were identified by combining data from multiple sources (self-report, medical records, medication use, biochemical measures, etc.), it is possible that some diagnoses were missed. It is unclear whether this misclassification differs according to exposure group or data source. Second, information on CR-related factors was collected from self-reports and may be subject to information bias. Third, the observed association might be biased by the established CR-dementia relationship. However, the associations remained after we removed dementia from the chronic disease outcome. Forth, there is the possibility of reverse causality, although results remained consistent in a sensitivity analysis excluding cases that occurred within the first two years of follow-up. Finally, healthy volunteer bias in the UK Biobank could limit the generalizability of our findings and may have contributed to an underestimation of the observed associations. Selection bias may be stronger in our sample as it was restricted to participants who were free from chronic diseases at baseline.

In conclusion, this study demonstrates that a high level of CR is associated with reduced risk of major chronic diseases and death and may prolong chronic disease-free survival, especially for people <60. Our findings also suggest that higher CR may buffer the adverse influence of smoking on chronic disease-free survival. Together, our results suggest that promoting CR-enhancing lifestyle experiences may be a feasible strategy to promote healthy longevity, especially for younger individuals and current smokers.

## Supplementary material

Supplementary material, approximately 410 KB.

## Data Availability

*Data Availability:* Access to UK Biobank data can be requested through a standard data access procedure. Requests to access these datasets should be directed to http://www.ukbiobank.ac.uk/register-apply.

## Notes

*Ethical standards:* The authors assert that all procedures contributing to this work comply with the ethical standards of the relevant national and institutional committees on human experimentation and with the Helsinki Declaration of 1975, as revised in 2013.
