# Supplementary material for: Association between Cognitive Reserve Indicator and Chronic Disease-Free Survival: A Large Community-Based Longitudinal Study
Source: J Prev Alzheimers Dis. 2024 Sep 18;11(6):1712–20. doi: 10.14283/jpad.2024.160 (PMC11573853; doi:10.14283/jpad.2024.160)
Supplement: Supplementary file 1 — Supplementary material, approximately 410 KB. [file 42414_2024_367_MOESM1_ESM.docx]

**Supplementary material**

**eMethod 1: Details on latent class analysis**

Several factors reflect different aspects of cognitive reserve (CR), including education, occupation, TV viewing time, frequency of confiding, frequency of social connection, and number of leisure activities, and thus we used these six variables to generate an overall CR indicator.

Latent class analysis (LCA) was used to create the CR indicator using the R LCA package. Likelihood ratio statistic *G*^2^, and Bayesian information criterion (BIC) were used for model selection, with lower values indicating a more reasonable model. We created a total of six latent class models, the details of which are reported in the following table.

**Table 1 for eMethod 1.** *G*^2^ statistics and BIC in models with different numbers of latent classes

| **Models** | ***G*^2^** | **BIC** |
| --- | --- | --- |
| One-latent-class model | 208003.6 | 6466006 |
| Two-latent-class model | 51417.32 | 6309665 |
| Three-latent-class model | 32208.56 | 6290702 |
| Four-latent-class model | 22598.73 | 6281338 |
| Five-latent-class model | 15432.13 | 6274417 |
| Six-latent-class model | 12601.49 | 6271832 |

In addition, to assess the uncertainty of posterior classification, we examined the mean posterior probabilities in models with three to six latent classes, with a value of 0.7 or more indicating an acceptable uncertainty. As shown in the table below, all mean posterior probabilities of the three-level solution are ≥ 0.7, therefore the three-level model is ideal for posterior classification uncertainty.

**Table 2 for eMethod 1.** Mean posterior probabilities in models with three to six latent classes

| **Models** | **Latent class 1** | **Latent class 2** | **Latent class 3** | **Latent class 4** | **Latent class 5** | **Latent class 6** |
| --- | --- | --- | --- | --- | --- | --- |
| One-latent-class model | 1.00 | - | - | - | - | - |
| Two-latent-class model | 0.91 | 0.89 |  |  |  |  |
| Three-latent-class model | 0.74 | 0.92 | 0.83 |  |  |  |
| Four-latent-class model | 0.69 | 0.73 | 0.82 | 0.76 |  |  |
| Five-latent-class model | 0.70 | 0.64 | 0.80 | 0.59 | 0.68 |  |
| Six-latent-class model | 0.65 | 0.57 | 0.60 | 0.63 | 0.78 | 0.62 |

In the three-latent-class model, *Latent class 1* had higher levels of education, occupational attainment, confiding, and leisure activities as well as less time spent watching TV. *Latent class 2* had lower levels of CR-related factors compared to *Latent class 1*. Additionally, there were overall low levels of CR-related factors in *Latent class 3*. Thus, *Latent class 1*, *Latent class 2*, and *Latent class 3* could be defined as “high CR indicator”, “moderate CR indicator”, and “low CR indicator”, respectively. The distribution characteristics of CR-related factors for the three-latent-class model are shown in the following table.

**Table 3 for eMethod 1.** Distribution characteristics of levels of cognitive reserve-related variables in three latent classes

| **Characteristics** | **Latent classes** | | |
| --- | --- | --- | --- |
|  | Latent class 1  (n=168211) | Latent class 2  (n=167766) | Latent class 3  (n=76532) |
| **Education level** |  |  |  |
| No educational qualifications | 0 | 1984 (1.18) | 60937 (79.62) |
| CSEs, O levels/GCSE or equivalent | 0 | 63105 (37.61) | 7892 (10.31) |
| A levels/AS levels, other professional qualifications or equivalent | 21720 (12.91) | 51161 (30.50) | 0 |
| NVQ, HND, HNC or equivalent | 6284 (3.74) | 51516 (30.71) | 6513 (8.51) |
| College/university degree | 140207 (83.35) | 0 | 1190 (1.55) |
| **Occupational attainment** |  |  |  |
| Unemployed or SEC 7 | 9406 (5.59) | 17652 (10.52) | 31847 (41.61) |
| SEC 4–6 | 10122 (6.02) | 39200 (23.37) | 26338 (34.41) |
| SEC 3 | 10955 (6.51) | 49005 (29.21) | 11318 (14.79) |
| SEC 2 | 66524 (39.55) | 43700 (26.05) | 4259 (5.56) |
| SEC 1.1 or SEC 1.2 | 71204 (42.33) | 18209 (10.85) | 2770 (3.62) |
| **Time spent watching TV (hours/day)** |  |  |  |
| ≥4 | 18433 (10.96) | 48602 (28.65) | 44509 (58.16) |
| 3–3.9 | 30371 (18.06) | 50294 (29.98) | 16309 (21.43) |
| 2–2.9 | 57025 (33.90) | 47259 (28.17) | 10276 (13.43) |
| <2 | 62382 (37.09) | 22151 (13.20) | 5348 (6.99) |
| **Frequency of confiding** |  |  |  |
| Never or almost never | 18052 (10.73) | 22660 (13.51) | 17447 (22.80) |
| About once a month or less | 18591 (11.05) | 18379 (10.96) | 8492 (11.10) |
| 1–4 times a week | 38822 (23.08) | 34607 (20.63) | 12964 (16.94) |
| Almost daily | 92746 (55.14) | 92120 (54.91) | 37629 (49.17) |
| **Frequency of social connection** |  |  |  |
| About once a month or less | 46145 (27.43) | 29572 (17.63) | 15451 (20.19) |
| About once a week | 64601 (38.40) | 61836 (36.86) | 23221 (30.34) |
| 2–4 times a week or more | 57465 (34.16) | 76358 (45.51) | 37860 (49.47) |
| **Richness of leisure activity engagement (/week)** |  |  |  |
| ≤0 | 41638 (24.75) | 49676 (29.61) | 32072 (41.91) |
| 1 | 70486 (41.90) | 75680 (45.11) | 34362 (44.90) |
| 2–5 | 56087 (33.34) | 42410 (25.28) | 10098 (13.19) |

Abbreviations: CSE, Certificate of Secondary Education; GCSE, General Certificate of Secondary Education; NVQ, National Vocational Qualification; HND, Higher National Diploma; HNC, Higher National Certificate; SEC, socio-economic classification.


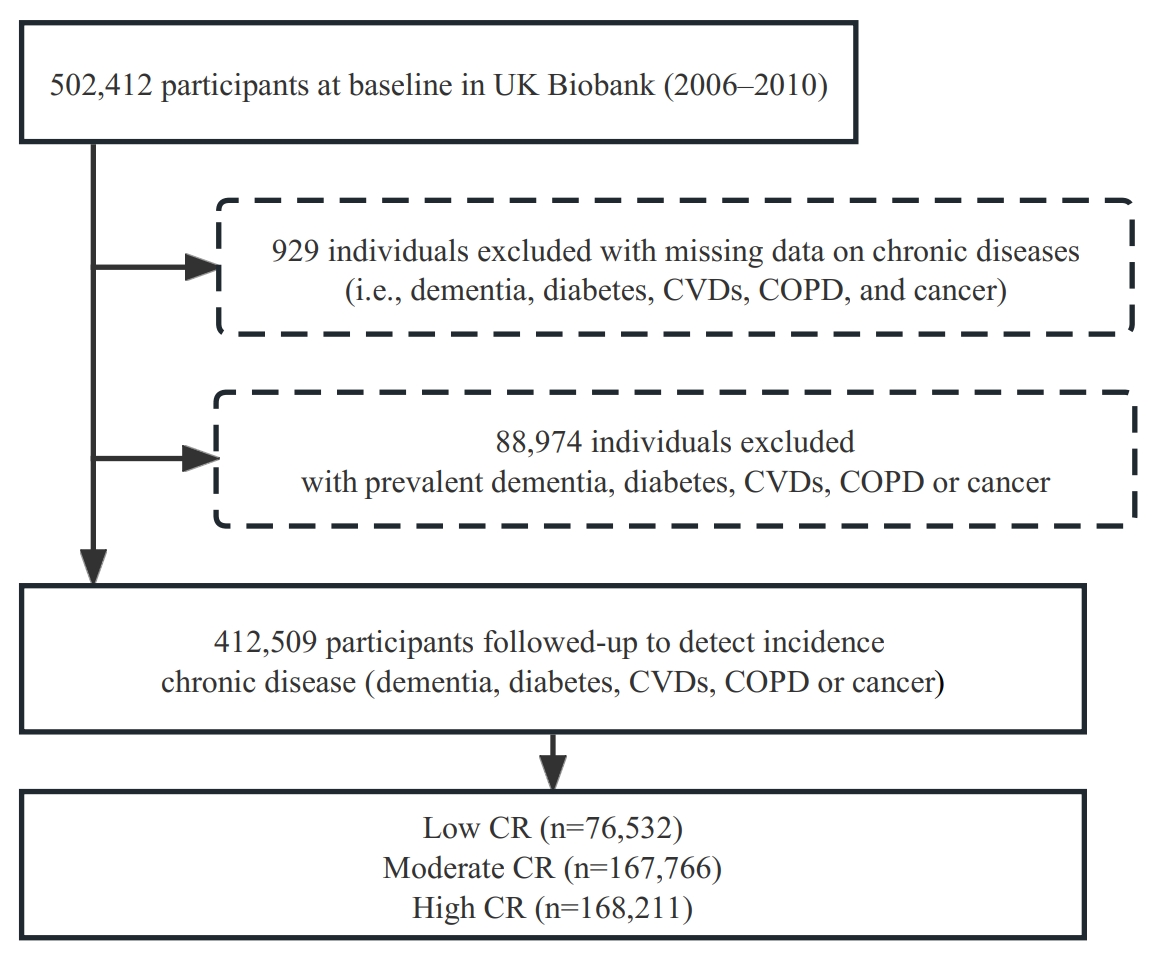
**eFigure 1** Flowchart of the study population

Abbreviations: CVDs, cardiovascular diseases; COPD, chronic obstructive pulmonary disease; CR, cognitive reserve


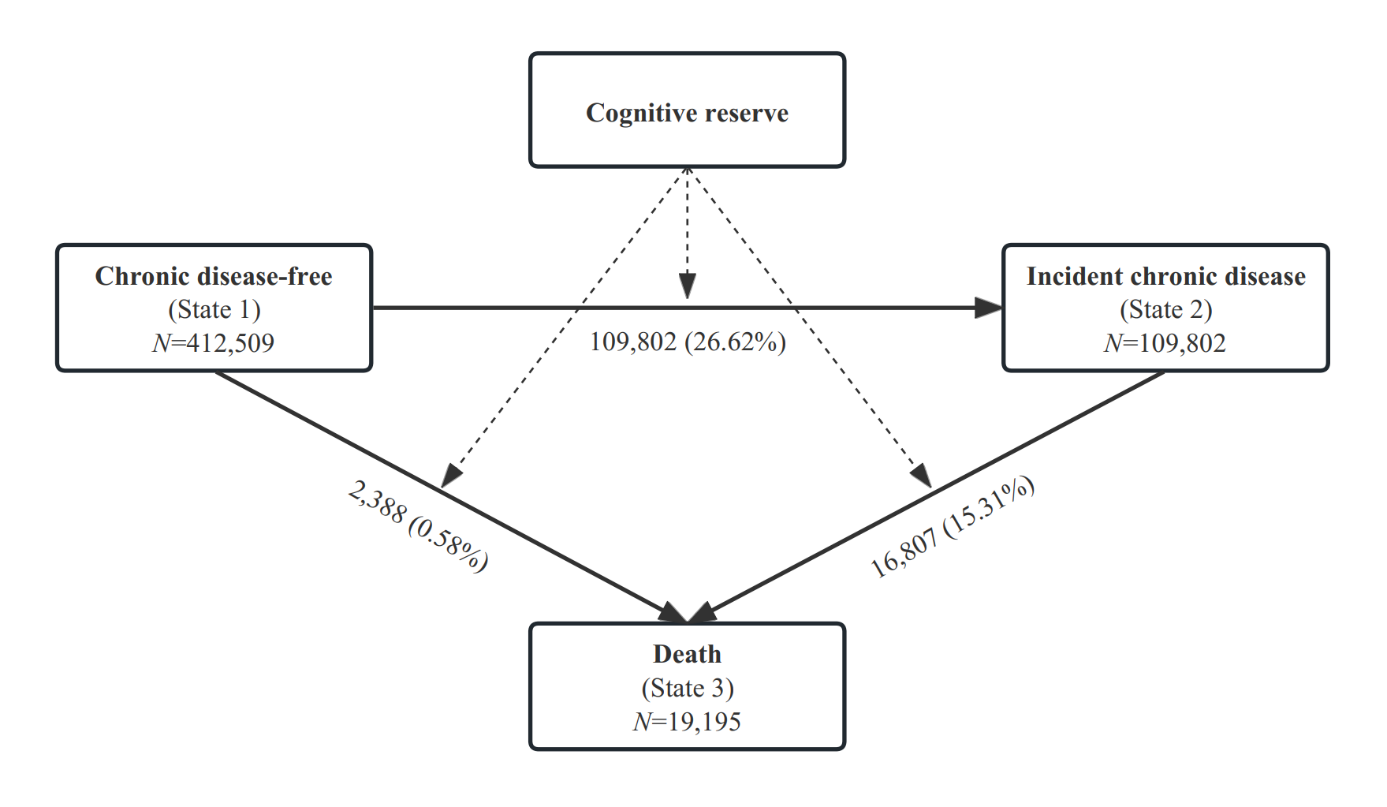
**eFigure 2** The multi-state models

**eTable 1** ICD-10 codes used to identify chronic disease

| **Chronic diseases** | **Codes** |
| --- | --- |
| Dementia | ICD-10 codes F00–03, F05.1, G30, G31.1, and G31.8 |
| Diabetes | ICD-10 codes E10-E14 |
| CVDs |  |
| Heart disease | ICD-10 codes I20, I25, and I48-I50 |
| Stroke | ICD-10 codes I60-I64 |
| COPD | ICD-10 codes J410-J44 |
| Cancer | ICD-10 codes C00-C97, except C44 for nonmelanoma skin cancer |

Abbreviations: ICD, international classification of diseases; CVDs, cardiovascular diseases; COPD, chronic obstructive pulmonary disease

**eTable 2** The 10th percentile differences (PDs) and 95% confidence intervals (CIs) in time (years) to death in relation to cognitive reserve indicator

| **Cognitive reserve** | **No. of**  **subjects** | **Death** | | |
| --- | --- | --- | --- | --- |
|  |  | No. of  cases | Basic-adjusted  10th PD (95% CI)^a^ | Multi-adjusted  10th PD (95% CI)^b^ |
| Low | 76532 | 6092 | 0.00 (Reference) | 0.00 (Reference) |
| Moderate | 167766 | 7092 | 2.04 (1.83, 2.26) | 1.47 (1.25, 1.68) |
| High | 168211 | 6011 | 2.82 (2.60, 3.04) | 1.80 (1.57, 2.03) |

^a^ Models were adjusted for age, sex, and race.

^b^ Models were further adjusted for smoking status, alcohol consumption, physical activity, body mass index, and hypertension.

**eTable 3** Hazard ratios (HRs) from Cox models and 33th percentile differences (PDs) in time (years) to incident outcomes (chronic disease or death) from Laplace regression, and 95% confidence intervals (CIs) in relation to cognitive reserve indicator, reported by dementia, CVDs, diabetes, COPD, or cancer separately.

| **Cognitive reserve** | **No. of**  **subjects** | **Chronic disease/Death** | | |
| --- | --- | --- | --- | --- |
|  |  | No. of cases | HR (95% CI) | 33th PD (95% CI) |
| **Dementia** | |  |  |  |
| Low | 76532 | 7153 | 1.00 (Reference) | 0.00 (Reference) |
| Moderate | 167766 | 8185 | 0.76 (0.73, 0.78) | 1.33 (1.16, 1.49) |
| High | 168211 | 6832 | 0.70 (0.67, 0.72) | 1.76 (1.58, 1.94) |
| **CVDs** | |  |  |  |
| Low | 76532 | 17721 | 1.00 (Reference) | 0.00 (Reference) |
| Moderate | 167766 | 24991 | 0.86 (0.85, 0.88) | 0.89 (0.76, 1.01) |
| High | 168211 | 21480 | 0.80 (0.78, 0.82) | 1.35 (1.21, 1.48) |
| **Diabetes** | | | | |
| Low | 76532 | 10731 | 1.00 (Reference) | 0.00 (Reference) |
| Moderate | 167766 | 13370 | 0.77 (0.75, 0.79) | 1.35 (1.20, 1.49) |
| High | 168211 | 10241 | 0.67 (0.65, 0.69) | 2.16 (2.00, 2.32) |
| **COPD** | | | | |
| Low | 76532 | 9938 | 1.00 (Reference) | 0.00 (Reference) |
| Moderate | 167766 | 10580 | 0.69 (0.67, 0.71) | 1.91 (1.76, 2.07) |
| High | 168211 | 7909 | 0.58 (0.57, 0.60) | 2.84 (2.68. 3.01) |
| **Cancer** | | | | |
| Low | 76532 | 14814 | 1.00 (Reference) | 0.00 (Reference) |
| Moderate | 167766 | 23483 | 0.93 (0.91, 0.95) | 0.44 (0.30, 0.57) |
| High | 168211 | 22063 | 0.92 (0.90, 0.94) | 0.46 (0.32, 0.60) |

Models were adjusted for age, sex, race, smoking status, alcohol consumption, physical activity, body mass index, and hypertension.

**eTable 4** Hazard ratios (HRs) from Cox models and 33th percentile differences (PDs) in time (years) to incident outcomes (chronic disease or death) from Laplace regression, and 95% confidence intervals (CIs) in relation to cognitive reserve indicator, stratified by age

| **Cognitive reserve** | **No. of**  **subjects** | **Chronic disease/Death** | | |
| --- | --- | --- | --- | --- |
|  |  | No. of  cases | HR (95% CI) | 33th PD (95% CI) |
| **Middle-age (<60 years)** | |  |  |  |
| Low | 32125 | 9011 | 1.00 (Reference) | 0.00 (Reference) |
| Moderate | 105203 | 20071 | 0.74 (0.72, 0.76) | 1.95 (1.78, 2.12) |
| High | 114687 | 18781 | 0.67 (0.66, 0.69) | 2.48 (2.30, 2.65) |
| **Old-age (60+ years)** | |  |  |  |
| Low | 44407 | 20098 | 1.00 (Reference) | 0.00 (Reference) |
| Moderate | 62563 | 24234 | 0.86 (0.84, 0.88) | 1.01 (0.86, 1.15) |
| High | 53524 | 19995 | 0.82 (0.80, 0.84) | 1.28 (1.12, 1.44) |

Models were adjusted for sex, race, smoking status, alcohol consumption, physical activity, body mass index, hypertension.

**eTable 5** Hazard ratios (HRs) from Cox models and 33th percentile differences (PDs) in time (years) to incident outcomes (chronic disease or death) from Laplace regression, and 95% confidence intervals (CIs) in relation to cognitive reserve indicator, stratified by smoking status

| **Cognitive reserve** | **No. of**  **subjects** | **Chronic disease/Death** | | |
| --- | --- | --- | --- | --- |
|  |  | No. of cases | HR (95% CI) | 33th PD (95% CI) |
| **Current smokers** | |  |  |  |
| Low | 11936 | 5710 | 1.00 (Reference) | 0.00 (Reference) |
| Moderate | 17926 | 5825 | 0.75 (0.72, 0.78) | 1.77 (1.52, 2.03) |
| High | 12683 | 3506 | 0.63 (0.61, 0.66) | 2.84 (2.55, 3.13) |
| **Never or previous smokers** | |  |  |  |
| Low | 64596 | 23399 | 1.00 (Reference) | 0.00 (Reference) |
| Moderate | 149840 | 38480 | 0.90 (0.88, 0.91) | 0.74 (0.62, 0.86) |
| High | 155528 | 35270 | 0.85 (0.83, 0.86) | 1.11 (0.99, 1.24) |

Models were adjusted for age, race, sex, alcohol consumption, physical activity, body mass index, and hypertension.

**eTable 6** Hazard ratios (HRs) from Cox models and 33th percentile differences (PDs) in time (years) to incident outcomes (chronic disease or death) from Laplace regression, and 95% confidence intervals (CIs) in relation to joint exposure of smoking status and cognitive reserve indicator

| **Joint exposure** | | **No. of subjects** | **Chronic disease/Death** | | |
| --- | --- | --- | --- | --- | --- |
| Smoking status | Cognitive reserve |  | No. of  cases | HR (95% CI) | 33th PD (95% CI) |
| Never or previous | High | 155528 | 35270 | 1.00 (Reference) | 0.00 (Reference) |
| Never or previous | Low | 64596 | 23399 | 1.19 (1.17, 1.21) | -1.12 (-1.25, -0.99) |
| Current | High | 12683 | 3506 | 1.41 (1.36, 1.46) | -2.26 (-2.50, -2.02) |
| Current | Low | 11936 | 5710 | 2.20 (2.13, 2.26) | -5.13 (-5.32, -4.93) |

Models were adjusted for age, sex, race, alcohol consumption, physical exercise, body mass index, and hypertension.

Measures of additive interaction between smoking status and cognitive reserve indicator on chronic disease or death:

RERI: 0.60, 95%CI: 0.53-0.68, *P*<0.001;

AP: 0.27, 95%CI: 0.25-0.30, *P*<0.001;

SI: 2.02, 95%CI: 1.83-2.23, *P*<0.001.

**eTable 7** Hazard ratios (HRs) from Cox models and 33th percentile differences (PDs) in time (years) to incident outcomes (chronic disease or death) from Laplace regression, and 95% confidence intervals (CIs) in relation to cognitive reserve indicator, stratified by sex

| **Cognitive reserve** | **No. of**  **subjects** | **Chronic disease/Death** | | |
| --- | --- | --- | --- | --- |
|  |  | No. of cases | HR (95% CI) | 33th PD (95% CI) |
| **Male** | |  |  |  |
| Low | 33162 | 14559 | 1.00 (Reference) | 0.00 (Reference) |
| Moderate | 67659 | 21425 | 0.87 (0.86, 0.89) | 0.91 (0.76, 1.07) |
| High | 78504 | 21615 | 0.81 (0.79, 0.83) | 1.46 (1.30, 1.62) |
| **Female** | |  |  |  |
| Low | 43370 | 14550 | 1.00 (Reference) | 0.00 (Reference) |
| Moderate | 100107 | 22880 | 0.86 (0.85, 0.88) | 0.98 (0.83, 1.13) |
| High | 89707 | 17161 | 0.82 (0.80, 0.84) | 1.31 (1.14, 1.47) |

Models were adjusted for age, race, smoking status, alcohol consumption, physical activity, body mass index, and hypertension.

**eTable 8** Hazard ratios (HRs) from Cox models and 33th percentile differences (PDs) in time (years) to incident outcomes (chronic disease or death) from Laplace regression, and 95% confidence intervals (CIs) in relation to cognitive reserve indicator, stratified by alcohol consumption

| **Cognitive reserve** | **No. of**  **subjects** | **Chronic disease/Death** | | |
| --- | --- | --- | --- | --- |
|  |  | No. of cases | HR (95% CI) | 33th PD (95% CI) |
| **Current drinkers** | |  |  |  |
| Low | 67804 | 25628 | 1.00 (Reference) | 0.00 (Reference) |
| Moderate | 156511 | 40972 | 0.87 (0.86, 0.88) | 0.91 (0.79, 1.03) |
| High | 158317 | 36921 | 0.82 (0.80, 0.83) | 1.33 (1.21, 1.45) |
| **Never or previous drinkers** | |  |  |  |
| Low | 8728 | 3481 | 1.00 (Reference) | 0.00 (Reference) |
| Moderate | 11255 | 3333 | 0.88 (0.83, 0.92) | 0.96 (0.61, 1.32) |
| High | 9894 | 2485 | 0.82 (0.77, 0.86) | 1.37 (0.99, 1.74) |

Models were adjusted for age, sex, race, smoking status, physical activity, body mass index, and hypertension.

**eTable 9** Hazard ratios (HRs) from Cox models and 33th percentile differences (PDs) in time (years) to incident outcomes (chronic disease or death) from Laplace regression, and 95% confidence intervals (CIs) in relation to cognitive reserve indicator, stratified by physical activity

| **Cognitive reserve** | **No. of**  **subjects** | **Chronic disease/Death** | | |
| --- | --- | --- | --- | --- |
|  |  | No. of cases | HR (95% CI) | 33th PD (95% CI) |
| **Physically inactive** | |  |  |  |
| Low | 13763 | 5613 | 1.00 (Reference) | 0.00 (Reference) |
| Moderate | 29760 | 8147 | 0.82 (0.80, 0.85) | 1.34 (1.08, 1.59) |
| High | 30424 | 7337 | 0.77 (0.75, 0.80) | 1.71 (1.44, 1.98) |
| **Physically moderate or active** | |  |  |  |
| Low | 62769 | 23496 | 1.00 (Reference) | 0.00 (Reference) |
| Moderate | 138006 | 36158 | 0.88 (0.87, 0.90) | 0.83 (0.71, 0.96) |
| High | 137787 | 31439 | 0.82 (0.81, 0.84) | 1.26 (1.14, 1.39) |

Models were adjusted for age, sex, race, smoking status, alcohol consumption, body mass index, and hypertension.

**eTable 10** Hazard ratios (HRs) from Cox models and 33th percentile differences (PDs) in time (years) to incident outcomes (chronic disease or death) from Laplace regression, and 95% confidence intervals (CIs) in relation to cognitive reserve indicator, stratified by body mass index

| **Cognitive reserve** | **No. of**  **subjects** | **Chronic disease/Death** | | |
| --- | --- | --- | --- | --- |
|  |  | No. of cases | HR (95% CI) | 33th PD (95% CI) |
| **BMI <25 kg/m^2^** | |  |  |  |
| Low | 20448 | 6867 | 1.00 (Reference) | 0.00 (Reference) |
| Moderate | 54857 | 12192 | 0.86 (0.83, 0.88) | 0.96 (0.74, 1.17) |
| High | 69800 | 13421 | 0.79 (0.77, 0.81) | 1.47 (1.26, 1.69) |
| **BMI 25+ kg/m^2^** | |  |  |  |
| Low | 56084 | 22242 | 1.00 (Reference) | 0.00 (Reference) |
| Moderate | 112909 | 32113 | 0.86 (0.85, 0.88) | 1.03 (0.90, 1.16) |
| High | 98411 | 25355 | 0.79 (0.78, 0.81) | 1.56 (1.41, 1.70) |

Models were adjusted for age, sex, race, smoking status, alcohol consumption, physical activity, and hypertension.

**eTable 11** Hazard ratios (HRs) from Cox models and 33th percentile differences (PDs) in time (years) to incident outcomes (chronic disease or death) from Laplace regression, and 95% confidence intervals (CIs) in relation to cognitive reserve indicator, by excluding participants with missing information (n=264,589)

| **Cognitive reserve** | **No. of**  **subjects** | **Chronic disease/Death** | | |
| --- | --- | --- | --- | --- |
|  |  | No. of  cases | HR (95% CI) | 33th PD (95% CI) |
| Low | 41964 | 12949 | 1.00 (Reference) | 0.00 (Reference) |
| Moderate | 104901 | 24787 | 0.87 (0.85, 0.89) | 0.92 (0.77, 1.07) |
| High | 117724 | 24236 | 0.81 (0.80, 0.83) | 1.35 (1.20, 1.51) |

Models were adjusted for age, sex, race, smoking status, alcohol consumption, physical activity, body mass index, and hypertension.

**eTable 12** Hazard ratios (HRs) from Cox models and 33th percentile differences (PDs) in time (years) to incident outcomes (chronic disease or death) from Laplace regression, and 95% confidence intervals (CIs) in relation to cognitive reserve indicator, by excluding participants who developed incident chronic disease or died in the first two years from baseline (n=398,524)

| **Cognitive reserve** | **No. of**  **subjects** | **Chronic disease/Death** | | |
| --- | --- | --- | --- | --- |
|  |  | No. of  cases | HR (95% CI) | 33th PD (95% CI) |
| Low | 72811 | 25388 | 1.00 (Reference) | 0.00 (Reference) |
| Moderate | 162263 | 38802 | 0.87 (0.85, 0.88) | 0.85 (0.74, 0.95) |
| High | 163450 | 34015 | 0.81 (0.80, 0.82) | 1.22 (1.11, 1.33) |

Models were adjusted for age, sex, race, smoking status, alcohol consumption, physical activity, body mass index, and hypertension.

**eTable 13** Hazard ratios (HRs) from Cox models and 33th percentile differences (PDs) in time (years) to incident outcomes (chronic disease or death) from Laplace regression, and 95% confidence intervals (CIs) in relation to cognitive reserve indicator, by excluding dementia from the chronic diseases

| **Cognitive reserve** | **No. of**  **subjects** | **Chronic disease/Death** | | |
| --- | --- | --- | --- | --- |
|  |  | No. of  cases | HR (95% CI) | 33th PD (95% CI) |
| Low | 76532 | 28715 | 1.00 (Reference) | 0.00 (Reference) |
| Moderate | 167766 | 43836 | 0.87 (0.86, 0.89) | 0.93 (0.82, 1.04) |
| High | 168211 | 38424 | 0.82 (0.81, 0.83) | 1.33 (1.21, 1.44) |

Models were adjusted for age, sex, race, smoking status, alcohol consumption, physical activity, body mass index, and hypertension.
